# Editorial: Secreted proteins as novel biomarkers and therapeutic targets in NAFLD

**DOI:** 10.3389/fendo.2023.1277101

**Published:** 2023-09-07

**Authors:** Xuelian Xiong

**Affiliations:** Zhongshan Hospital, Fudan University, Shanghai, China

**Keywords:** secreted protein, biomarker, NAFLD, NASH, therapeutic target

Non-alcoholic fatty liver disease (NAFLD) is a prevalent liver condition, closely linked to metabolic syndrome (1). Its global occurrence has surged to 25% in the past decades in tandem with rising obesity and type 2 diabetes rates (2). NAFLD encompasses a spectrum of hepatic states, spanning from nonalcoholic fatty liver (NAFL) to nonalcoholic steatohepatitis (NASH), with elevated risks of progressing to cirrhosis or even hepatocellular carcinoma (HCC) (3, 4). NAFLD patients often exhibit no symptoms, and their manifestation typically indicates more advanced liver disease or unrelated complications (5). Presently, liver histology remains the benchmark for diagnosing and staging NAFLD. However, the challenges of repeated liver biopsies, due to health risks, individual variations, sampling inaccuracies, and high costs, persist (6).

The substantial economic and healthcare burden of NAFLD is partially attributed to its high incidence and the absence of early biomarker detection (7). As defined by the FDA, a biomarker is “an objectively measured characteristic that is measured as an indicator of health, disease, or a response to an exposure or intervention, including therapeutic interventions” (8). The goal of the biomarker field is to establish swift, dependable, cost-effective, and potent detection and monitoring techniques for early NAFLD identification and assessing HCC risk. This way, physicians can track disease advancement, and patients can receive optimal therapies.

Multiple pathways and interactions, modulated by endocrine factors and metabolites, connect various organs and cells, influencing the pathogenesis of NAFLD. Secreted proteins, acting as crucial mediators, play a pivotal role in this pathogenesis (7). Altered protein expression is frequently tied to specific diseases (9). Prior research has highlighted that secreted proteins offer inherent advantages for diagnosing and treating clinical conditions. Hepatokines, for instance, secreted by hepatocytes, can impact metabolic processes (10). Yang et al. through ingenuity pathway analysis (IPA), explored signaling pathways and interactions among muscle, adipose tissue, hepatic stellate cells, and other liver cells. They classified the interplay between hepatokines, myokines, and adipokines in NAFLD and NASH. Importantly, these mediators, including liver-secreted proteins, influence not only the liver’s metabolic processes but also entire physiological systems (11). Earlier studies have revealed that up to 75% of chronic liver disease patients display osteoporosis symptoms (10). Zhao et al. concentrated on the liver-bone axis, summarizing the latest research to underscore the significant correlation between NAFLD and osteoporosis. They examined several liver-secreted endocrine factors and metabolites involved in bone metabolism regulation, encompassing IGF-1, FGF21, IGFBP1, fetuin-A, TNF-α, and OPN. Nonetheless, the therapeutic potential of these pathways, interactions, and mediators has yet to be effectively translated into clinical practice.

Uric acid has been identified as a regulator of hepatic steatosis and insulin resistance via inflammasome pathways (12). High-density lipoprotein cholesterol (HDL-C), known for its anti-inflammatory and antioxidant functions, constitutes a plasma lipoprotein (10). Elevated uric acid levels (hyperuricemia) and decreased HDL-C levels significantly correlate with a heightened risk of NAFLD (13). In a cross-sectional study encompassing 3,766 participants, Xie et al. investigated the correlation between the uric acid-to-high-density lipoprotein cholesterol ratio (UHR) and the likelihood of NAFLD, along with the severity of liver steatosis and fibrosis. They identified that increased UHR levels were independently associated with an elevated NAFLD risk and the extent of liver steatosis in the American population.

Regulated cell death plays a critical role in metabolic liver disease outcomes. Various types of hepatocyte death trigger a cell-cell network that drives the progression of metabolic liver disease toward inflammation, fibrosis, and, eventually, cirrhosis. A recently identified form of regulated cell death, known as ferroptosis, also contributes to inflammatory responses and activates hepatic stellate cells (14), further exacerbating NAFLD progression. Through bioinformatics and a literature-based ferroptosis database, He et al. identified ferroptosis-related genes involved in NASH. Their findings revealed a significant association between the expression of ferroptosis-related genes, such as CDKN1A and SIRT1, and the progression of fibrosis in NASH.

Biomarkers hold promise for expediting drug development by serving as early indicators of improved clinical responses, enhancing patient safety, and enabling personalized medicine. Secreted proteins, acting as non-invasive indicators, could significantly bolster the ability to anticipate the onset of NAFLD and reveal potential targets for therapeutic intervention. The articles in the Research Topic “*Secreted proteins as novel biomarkers and therapeutic targets in NAFLD*” offer valuable insights into new secreted proteins and metabolites that could serve as serum biomarkers or potential pharmacological candidates for NAFLD. These examples underscore the increasing relevance of multidisciplinary, technology-driven NAFLD diagnostics as an alternative to traditional techniques. Nevertheless, there remains a considerable need to establish and validate additional biomarkers or emerging technologies capable of predicting NASH progression and treatment response.

## Author contributions

XX: Writing – original draft, Writing – review & editing.
